# Organizational factors associated with Health Care Provider (HCP) influenza campaigns in the Veterans health care system: a qualitative study

**DOI:** 10.1186/s12913-016-1462-y

**Published:** 2016-07-04

**Authors:** Zayd Razouki, Troy Knighton, Richard A. Martinello, Pamela R. Hirsch, Kathleen M. McPhaul, Adam J. Rose, Megan McCullough

**Affiliations:** Center for Health Services Research in Primary Care, Durham VA Medical Center, Durham, NC 27705 USA; Department of Veterans Affairs, Office of Public Health, Washington, DC USA; Center for Health care Organization and Implementation research, Bedford VA Medical Center, Bedford, MA USA; Department of Medicine, Section of General Internal Medicine, Boston University School of Medicine, Boston, MA USA; Departments of Internal Medicine and Pediatrics, Yale University, School of Medicine, New Haven, CT USA

**Keywords:** Health care provider, Influenza vaccine, Organizational factors, Positive deviance method, Qualitative study

## Abstract

**Background:**

It is an important goal to vaccinate a high proportion of health care providers (HCPs) against influenza, to prevent transmission to patients. Different aspects of how a HCP vaccination campaign is conducted may be linked to different vaccination rates. We sought to characterize organizational factors and practices that were associated with vaccination campaign success among six sites within the Veterans Health Administration, where receipt of flu-vaccination is voluntary.

**Method:**

We conducted a total of 31 telephone interviews with key informants who were involved with HCP flu vaccination campaigns at three sites with high-vaccination rates and three sites with low-vaccination rates. We compared the organization and management of the six sites’ campaigns using constant comparison methods, characterzing themes and analyzing data iteratively.

**Results:**

Three factors distinguished sites with high flu vaccination rates from those with low vaccination rates. 1) High levels of executive leadership involvement: demonstrating visible support, fostering new ideas, facilitating resources, and empowering flu team members; 2) Positive flu team characteristics: high levels of collaboration, sense of campaign ownership, sense of empowerment to meet challenges, and adequate time and staffing dedicated to the campaign; and 3) Several concrete strong practices emerged: advance planning, easy access to the vaccine, ability to track employee vaccination status, use of innovative methods to educate staff, and use of audit and feedback to promote targeted efforts to reach unvaccinated employees.

**Conclusion:**

Successful HCP flu campaigns shared several recognizable characteristics, many of which are amenable to adoption or emulation by programs hoping to improve their vaccination rates.

**Electronic supplementary material:**

The online version of this article (doi:10.1186/s12913-016-1462-y) contains supplementary material, which is available to authorized users.

## Background

While there is support for mandating influenza vaccination for health care providers, there are also dissenting opinions [1, 2]. This debate has been fueled by important organizations on both sides, including American College of Occupational and Environmental Medicine (ACOEM) and the Society for Healthcare Epidemiology of America (SHEA). The ACOEM argues that the evidence of benefit is not sufficient to override employees’ autonomy to refuse the vaccine [3], while SHEA views HCP vaccination as a core patient safety practice and noncompliance should not be tolerated [4]. The ACOEM published a guidance statement emphasizing the importance of educating, publicizing and offering the flu vaccine at convenient times and places as a way to achieve a high vaccination rate, instead of relying upon a mandate [3]. This emphasis underscores the importance of understanding organizational factors that impact the success of HCP-flu campaigns, especially when a mandate is not feasible [5, 6]. While previous studies have explored some of the organizational factors that may influence flu campaign performance in terms of achieving high vaccination rates, [7–10], much remains to be learned about this topic. We sought to further explore and describe organizational these factors.

Our study was commissioned by the Veterans Health Administration (VHA) Office of Public Health. VHA is the largest integrated healthcare system in the United States, and employs 321,000 healthcare workers. In the VHA, employee receipt of flu-vaccination is voluntary. Like other healthcare systems, some sites within VHA consistently achieve high rates of employee influenza vaccination and some sites consistently achieve low rates. We aimed to identify organizational characteristics present at sites with high vaccination rates, using sites with low rates for the purpose of comparison. Our results can be used to inform efforts to create more effective HCP vaccination campaigns, and by extension help improve other safety initiatives that target HCP behavior.

## Methods

### Selection of study sites

We used a study design called the “positive deviance method” [11]. The positive deviance method is an increasingly common design study used in the fields of public health and health services research. Outliers, in this case medical sites with extremely high or extremely low HCP flu vaccination rates, are examined in depth using qualitative research methods. The Positive Deviance method focuses on identifying successful practices that already exist at sites with high performance (i.e. sites with extremely high HCP vaccination rates). Low performance sites (i.e. sites with extremely low vaccination rates) are included to provide contrast, and help distinguish which practices differentiate between high and low performing. The open-ended interview questions also enables the collection of rich data regarding context and unanticipated findings. After identifying successful practices which are consistently found at high-performing sites and absent at low-performing sites, the next step is to promote their use more widely. A strength of the Positive Deviance method is that the strong practices identified are by definition practical, as they are already successfully employed by some sites.

We selected our study sites based on their HCP vaccination rates in three consecutive fiscal years (2011–2013). Our three high-performing sites consistently had HCP vaccination rates higher than 70 %, while our three low-performing sites consistently had vaccination rates below 40 %. We reasoned that consistent exemplary or poor performance could best reveal organizational elements that impact vaccination rates and ensure we included sites based on a durable pattern of performance.

We extracted the vaccination rates from a central database that we obtained from the VA Clinical Public Health Group. We also matched high and low performing sites according to the facility size (the number of employees working at each facility). Basic descriptive information about sites is shown in Table 1.

**Table 1:** Site characteristics for six Veteran Health Administration Medical Centers

Site code	Vaccination rates from 2011–2013	Geographical location	Size facility	Number of conducted interviews at each site
1-	High- 73 %, 89 %, 71 %	Northwest	Large: 5000 employees	Eight
2	High- 80 %, 82 %, 98 %	Mid-West	Medium: 1100 employees	Six
3	High- 74 %, 76 %, 97 %	Mid-Atlantic	Small: 800 employees	Three
4	Low- 37 %, 40 %, 41 %	Northeast	Small: 800 employees	Four
5	Low- 38 %, 38 %, 31 %	Mid-Atlantic	Medium: 1100 employees	Three
6	Low 26 %, 26 %, 28 %	Southwest	Large: 6000 employees	Seven

Number of employees is approximate, but accurate within 10 %

### Data collection

The Institutional Review Board (IRB) at the Bedford VA Medical Center exempted the study from review because it was considered quality improvement work. We were not required to obtain a formal written consent, but we did obtain verbal agreement from all participants, and the elements of informed consent were present. This included informing pariticpants about the purpose of the study and who was sponsoring it, that participation was voluntary, that their identities would be kept confidential, and risks and benefits to participants.

We recruited sites with a letter sent from the VHA Office of Clinical Public Health requesting their participation in our study. This letter described the aim of the project, namely to examine organizational-level factors contributing to HCP flu campaigns. We did not inform sites or participants of their high- or low-outlier status. Each participating site identified a point of contact that assisted in identifying staff who are involved in the local HCP flu campaign. We also interviewed relevant leadership figures at each site, such as the Chief of Staff, to assess their support for the campaign. No site declined our invitation to participate in the study, and no staff member at any of the included sites declined to participate. See Table 2 for the professional roles of interviewees.

**Table 2:** Roles of staff interviewed at Study sites (18 staff members from high-performing sites and 13 members from low-performing sites)

Roles of staff interviewed at study sites	Number of interviews
Senior Leadership (Director, Deputy Director, Chief of Staff)	6
Flu Coordinator	6
Occupational Health Department (Physician and Nurse)	6
Infection Control Department (Nurses or Hospital Epidemiologist)	7
Health Promotion and Disease Prevention Coordinator or Employee wellness Coordinator	3
Nurse Educator	1
Union official	1
Nurse Manager for affiliated Community Living Center (also known as a Skilled Care Facility	1
Total	31

Two authors (ZR and TK) jointly conducted 31 telephone interviews, from June 2013-March 2014. Interviews were recorded and transcribed verbatim. Interviews ranged in duration from 20–60 min. We interviewed staff individually in most cases; but sometimes interviewees chose to speak to us in pairs.

Semi-structured interviews aim to explore what people say in as much detail as possible thereby uncovering new areas or ideas that were not anticipated at the outset of the research [12] The inclusion of open-ended questions provides guidance and focus for the interview on the topic at hand and yet interviewers are able to follow topical trajectories in the conversation when the interviewer feels this is appropriate [13]. Semi-structured interviewing with an interview guide provides reliable, comparable qualitative data [13].

We used an interview guide that our study group developed prior to starting the interviewing process (Additional file 1). Topics included asking participants to describe their role and the role of others in the flu campaign. We also asked participants to describe how HCP vaccine campaigns are planned, promoted, executed and evaluated at their facility.

### Data analysis

Interviews were coded with pre-defined concepts, which were based upon the ACOEM consensus statement about HCP flu campaigns. Concepts included leadership role, feasibility of accessing the vaccine, resources available to the campaign, and implementation strategies. Three authors (ZR, AJR, and MM) coded transcripts independently and then met to discuss coding. By our third interview with each site, we noticed considerable repetition of themes, suggesting that we had reached thematic saturation [14]. Interviews were analyzed using the constant comparative method, whereby pre-defined codes were refined and new codes were identified and elaborated in an iterative fashion based on analysis of the interviews and coding discussions [15]. Coding discrepancies were resolved by in-depth discussion and negotiated consensus. We then created a profile for each site that organized recurrent concepts under major headings, summarizing data gleaned from all interviews and sites. The most important findings in a positive deviance study are those practices that are consistently present among high performing sites but are absent (or at least less pronounced) among low performing sites. In addition, this approach allowed us to unravel factors that were simply unique to a single site. The methodology and data analyses of this manuscript in its current format addresses all relevant items included in RATS checklist for reporting qualitative studies.

Results were shared with coauthors from the VA Clinical Public Health Group, who have direct experience organizing flu campaigns, to ascertain face validity of findings.

## Results

### Overview

**Fig. 1:**
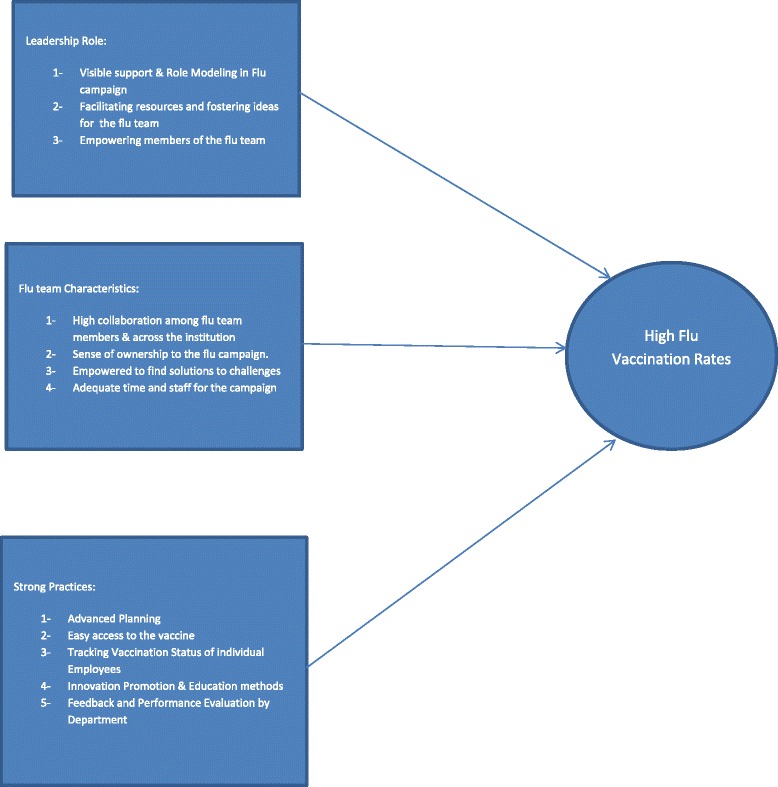
Organizational Factors that are associated with high flu vaccination rates

**Fig. 2:**
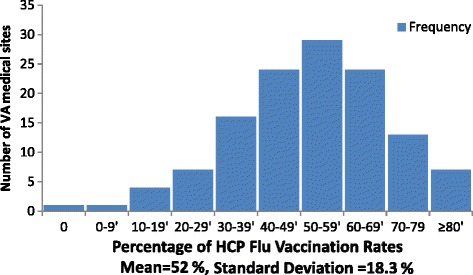
Depicts the distribution of HCP Influenza Vaccination rates for (*N* = 126) VA Medical Centers

We show the overall performance of VA sites regarding vaccinating employees in Fig. 1. We refer to the collective efforts of the different stages to provide the flu vaccine to employees as the “flu campaign”. Also, since all sites implement their “flu campaigns” using a team structure, we refer to all members who are involved as the “team”. We will briefly summarize our main findings here, and will explain in detail in the remainder of the results section. Three factors distinguished sites with high flu vaccination rates from those with low vaccination rates. First, executive leaders were more highly involved in the flu campaign at high outlier sites. They demonstrated visible support, fostered new ideas, facilitated resources, and empowered flu team members. Second, flu teams at sites with high vaccination rates had positive characteristics that included high levels of collaboration among the flu team and across the institution, a sense of campaign ownership, a sense of empowerment to meet challenges, and adequate time and staffing dedicated to the campaign. Third, successful campaigns shared certain strong practices that included advance planning, proactive efforts to make the vaccine accessible to employees, an ability to track employee vaccination status, the use of innovative methods to reach employees with education and promotion, and a use of performance data to guide the ongoing campaign in real time. See Fig. 2 for a summary of these three main findings, which will be described in detail below.

As a further orientation to the reader, Sites 1–3 are high-performing sites, while Sites 4–6 are all low-performing sites. For each organization factor that we identified, we will present findings about high-performing sites first, and then contrast it with our observations from low-performing sites. Italics in quotations signify emphasis by the speaker.

### Organizational factor 1: executive leadership involvement

One of our most consistent findings was that executive leadership figures, such as the Chief of Staff, were much more involved with the campaign at the high outlier sites. Their role was pivotal in ensuring the success of the campaign.

At high performing sites, leadership support for the campaign was highly visible, and contributed to a general perception of the campaign being an important priority. For example, some leaders at successful sites engaged in “role modeling” where they demonstrated their support for the campaign by volunteering to administer the vaccine to employees. One interviewee at Site 1 said, “[The nurse executive] makes it a point to volunteer every year to do that”. Leadership figures at Site 3 also participated in giving flu shots. They explained that they do this “So the staff gets the message that it’s important to the leadership to do this.”

At some sites, executive leadership members communicated the importance of the campaign in even more innovative and sometimes lighthearted ways. At Site 2, the Chief of Staff acted in a skit presented to medical center staff which promoted the campaign.

Leadership figures at high-performing sites not only supported the campaign symbolically, but were also involved directly in the campaign in substantive ways. Leadership members secured prime sites to offer the vaccine, authorized symbolic incentives (such as an extra hour of leave) for employees who get vaccinated, and were likely to support new ideas proposed by the flu team. Additionally, at high performing sites, the flu team held frequent meetings with executive leadership throughout the flu vaccination “season” (the autumn) and shared with them the campaign’s progress and needs.

In addition, leadership empowered the flu team and they offered them a wide platform to communicate the importance of the campaign and its progress through town hall meetings and other large events attended by large audiences. For example, at Site 2, leadership figures agreed to offer the vaccine at the staff fall picnic party.

In contrast to the above, there was a lack of visible leadership support at low performing sites. This lack of visibility de-emphasized the flu campaign’s importance as an institutional goal. At Site 5, a low performing site, one interviewee said,

“In short, I think it’s institutional will as well, I mean if the Director says he wants it done then people listen, if the Chief of Staff asks her secretary to send out a notice that there’s going to be employee flu clinic tomorrow then the employees get the notice… [In the absence of this kind of public emphasis]…the employees get the message ‘It’s not that important’… I can guarantee you when the [new unit] reopens at the end of next month, there will be balloons and banners and notices every hour to come by and visit it, and the Director will go there and the Chief of Staff will go there and everyone will know that they consider it to be very important.”

Similarly, the infection control nurse at the same site said “It would be nice to see the Director or the Director of Nursing going out there and encouraging her staff to get the flu vaccine. That would impress me as a staff nurse.”

Furthermore, at low- performing sites, leadership did not remove barriers encountered by the team. At low performing sites, campaigns were beset with red-tape, which resulted in barriers such as difficulty advertising the campaign with promotional banners. One team member (Site 4) remarked,

“I will *also* say very *frankly* that …there hasn’t*…* been the kind of interest that I would like to see from administration about this …I mean it’s mostly a question of can you [leadership]…put some money towards this. And there hasn’t been interest in that…I…suggested could we make a banner, a really nice banner that isn’t…time sensitive…Ya know, ‘Get Your Flu Shots Now!’ that kinda thing and…the response wasn’t like, yeah, great idea”.

In contrast to high performing sites, where leaders approved small incentives for employees to be vaccinated, at Site 6, a suggestion to incentivize employees to get the flu vaccine was rejected by leaders. When asked why, the flu-coordinator explained, “…the leaders of Ambulatory Care; they said, well,…that would require us to organize; we would have to get our staff to make sure that everybody had extra work…and they didn’t want to do it”. A nurse reported difficulties securing a place to give the vaccine “I gotta say we couldn’t even get the room last year because of the basketball game.” [Infection control nurse, Site 5].

Team members at low-performing sites conducted infrequent meetings with leadership, sometimes only at the end of flu season, or no meetings at all, so that there was little involvement of leadership and few opportunities for leaders to help with problem-solving; the flu vaccination team members were generally not empowered to make decisions or take actions to meet the campagins changing needs.

### Organizational factor 2: positive flu team characteristics

We consistently observed that flu team members from the high-performing sites worked together closely and communicated often. For example, the occupational health nurse at Site 1 said “I always keep [the infection control nurse] in the loop, so if she needs something…she just emails [me] directly. So…we just work together *really* closely on a day in, day out basis.” The infection-control nurse at Site 2 said, “we’re [the flu team] pretty much a well-oiled machine…everybody knows their duties” At Site 3, one interviewee said “[my colleague] and I work *really* well together. She… does a lot of phone calls for me. She will collect data for me…she’ll send out…emails for me as well.” In addition, the flu team at high performing sites established close working relationships with key people across the institution, such as department managers, nursing shifts supervisor, and other flu team liaisons. These contacts contributed significantly to the success in many aspects of the flu campaign, including delivering the vaccine, tracking and reporting employees’ flu vaccine status, and publicizing opportunities to receive the vaccine.

At high performing sites, team members felt a sense of “ownership” over the flu campaign, and served as its personal representatives to front-line staff. At Site 3, the occupational health nurse said, “So basically, my *whole* idea is, I have a sense of ownership of this…it’s like *mine* and I feel that, I own it, so I focus this energy on getting the results I want…”. Similarly, at Site 2, the education nurse proudly associating her figure to the flu campaign said that she is called “the flu queen” at her institution. She said, “I mean, I’m sure people when they see me in the hall, they picture me as a walking flu shot”.

Additionally, team members at high-performing sites were creative, and felt empowered to find solutions to challenges. They were characterized by a tenacious effort that often required going beyond the call of duty. One member of the flu team at Site 2 stated that her colleagues’ efforts on behalf of the campaign made it into a great success: “…the hard work that our employee health nurse puts into it and our health promotion disease prevention person puts into it. They really get out there and they talk with staff and they try to promote that vaccine.”

Finally, high-performing sites had the flexibility to dedicate staff and time for the campaign, allowing them to spend most of their time on the campaign during the flu-vaccination season. When we asked an occupational health nurse at Site 3 about how she manages her time during the flu-season, she replied,“… on my schedule, I’ll block off like one day a week or two days a week. Those are my flu days. So no one can schedule *anything* on those days, and then the other three days of the week you can schedule my pre-employment physical exams…”

In contrast, at low-performing sites, teamwork was generally absent. At most low performing sites, one department or one person was responsible for the entire campaign, while others served in a peripheral role. The infection-control nurse at Site 4, when asked how much time she spent on the campaign, replied, “No, not much at all. I wouldn’t have even put it down on my resume were I to write a new one. I’m just consulting with occupational health.” Similarly, at Site 6, the flu-coordinator said, “…we’re distant from each other and it’s sometimes hard to organize a system-wide campaign because…there are so many disparate parts”.

Sometimes, team members expressed frustration at being asked to run the flu campaign alone, and wished that others would be more involved. The flu-coordinator at Site 6, speaking of infection control, said, “I really believe they could’ve been more involved but they are consultative only, according to them.” Similarly, at Site 4, the occupational health nurse said: “I don’t think it’s seen as a hospital-wide responsibility. I don’t think the culture here is, that it’s really…a big *subject* for some reason.” In contrast to high-performing sites, where flu team members had strong connections and working relationships not only with each other, but also with key contacts across the organization, such contacts were absent at low-performing sites.

Furthermore, in contrast to the high-performing sites, we observed a lack of a sense of “ownership” of the campaign by team members at lower performing sites. At Sites 5 and 6, we found that there was no consensus about who should spearhead the HCP flu campaign. At Site 5, the person formally assigned to champion the HCP flu campaign was not aware of his role. At Site 6, the assigned nursing department for this task did not view the HCP flu campaign as part of their role. The flu-coordinator at that site described it as, “this flu campaign was kind of the [unwanted project]. Nobody wanted to do it.” She also noted that there is a lack of accountability when assigning a task, leaving the burden of achieving the task on one person.

“…, we did have [a] department accept a task at one point in time…I recall just having to stay after them *week* after *week* after *week* to say is *this* done, is this *done*? And it never got done, so I ended up picking up and doing it. …so it’s the accountability piece of it and making sure that one person is not just the only individual running around and working.”

In contrast to the positive attitude of team members at high-performing sites to manage the flu campaign, team members at low-performing sites focused on external factors as explanations for their low vaccination rates, such as the culture of their geographical region, mild flu-seasons, and the absence of a mandating policy for the flu vaccine. There was a sense of helplessness; respondents had difficulty imagining what could improve their performance. The Chief of Staff at Site 5 said:

“…it’s really hard to single handedly overcome public perception which I think is really what we’re looking at here. There’s a high percentage of the public population that don’t get the flu shot when the flu’s not around, and they think they get the flu when they get the shot so, I think this is a nationwide issue, not just a VA issue. So until we can mandate [that all employees must be vaccinated], I think it’s gonna be really hard.”

This participant identifies several possible explanations for why his site’s campaign is doing poorly year after year; tellingly, none of these factors is amenable to change.

While at high sites, the flu team was had the flexibility to spend considerable time and effort on their campaign, especially during the vaccine season, at low-performing sites, team members were expected to continue the same work productivity during the vaccine season as they do the remainder of the year. The flu-coordinator at Site 6 said:

“I do a lot of things. I’m in charge of the inpatient performance measures. I’m in charge of the…nurse practitioner practice here. We have a hundred and five NP’s here. Uh, this is a collateral assignment. I also hold a panel of patients in cardiology.”

When we asked if she can designate time to work on the flu campaign, she responded, “I can’t, because I just look at what’s a priority and I go with it… ”

Similarly, members of the flu team at low-performing sites felt the amount of staff effort devoted to the flu campaign was inadequate. The hospital epidemiologist at Site 5 said, “There is always a tug and a pull between what they’re doing and what they are able to do, and I do think that they [the flu team] are understaffed for a hospital of this size”. The flu Manager at Site 6 stated, “We don’t have designated staff to give vaccination. It’s *whoever’s* on light duty; whoever has a foot that’s in a cast and can’t really walk, whoever’s pregnant…flu is not considered a very high priority by this institution” [italics for emphasis].

### Organizational factor 3: strong practices

Each site’s HCP flu campaign varied based on various idiosyncratic factors, such as the number of off-site campuses, the geographic size of the campus, and the number of HCP’s that had to be reached, among other issues. However, despite these local idiosyncrasies, we identified 5 strong practices that were consistenly present at high sites and absent at low sites.

### Strong practice 1: advance planning

High-performing sites did more advance planning in preparation for the flu vaccination season. Sites 1 and 2 used hospital-wide events, such as emergency drills and annual picnics, as an opportunity to reach large numbers of employees. Extensive advance planning was required to set locations and schedules in partnership with different departments at their institution. As another example of strong advance planning, Site 2 developed an action plan detailing the role of each team member in the flu campaign. This plan serves as a guide to assign tasks that need to be accomplished by a predetermined deadline. It includes times for regular, fixed meetings and a reference for troubleshooting problems. In contrast, this sort of detailed and proactive advance planning was absent at the low performing sites.

### Strong practice 2: making the vaccine easy to access

High-performing sites had multiple avenues for delivering the vaccine for longer periods of time. For example, Site 1 provides walk-in clinics that are open 3-4 times a week, including weekends, from 9:00 am to 9:00 pm, from October through December. Also, the flu vaccine is offered during an annual emergency drill for one week, from 9:00 am–9:00 pm. Departments participate in the drill according to pre-arranged times, but individual employees can also attend and get vaccinated. Moreover, flu “blitzes” are scheduled and coordinated between the occupational health nurse and designated “health liaisons” for off-site campuses. Site 2 similarly offers the flu vaccine during the fall staff picnic time. They also seize unscheduled opportunities to offer the vaccine using time scraps, announcing their availability and location using the hospital address system.

Low performing sites offered the vaccine in a less frequent and intense manner compared to high performing sites. In addition, low performing sites did not integrate their campaigns with other large hospital activities.

Some modalities of vaccine delivery appeared to be used both by high- and low-performing sites, such as walk-in clinics, staff vaccinating each other, offering the vaccine at large staff meetings and using the rolling carts. Because they were present at both high and low sites, these modalities did not appear to distinguish between high and low performing sites. Indeed, these modalities were the only ones employed at the low performing sites, as well as site 3. However, Site 3 offered them more frequently and over longer time periods, which presumably contributed to the improved results.

### Strong practice 3: tracking vaccination status of individual employees

All facilities had access to an electronic system that allows them to monitor vaccination rates at the instituational level. However, only high-performing sites had effective processes to track the vaccination status for each individual employee. A record was kept and updated for individual employees who received the vaccine or declined it. High-performing sites actively encouraged employees to report their vaccination status, so that they could enter it into this tracking system. Also, combining the flu vaccine with a mandatory emergency drill provided a convenient opportunity to collect employees’ vaccination status en masse. Following that event, flu team members at Sites 1 and 2 contact local managers, who then assume the responsibility to contact employees whose vaccination status remains unknown and communicate that status to the flu team. This process was repeated weekly or bi-weekly throughout the vaccination season. Similarly, at Site 3, the occupational health nurse generates a list of employees and tracks employees whose vaccination status remains unknown. This process was reinforced by a local policy that requires signing a declination form by those who refuse the vaccine.

Low performing sites lacked any organized process to track employees’ vaccination status. Without knowing the vaccination status for employees, it was difficult to target extra efforts to those who had declined the vaccine or who simply had not had an opportunity to receive it.

### Strong practice 4: innovative promotion and education methods

At high-performing sites, the team invested time and effort in educating staff about the flu vaccine, even if they ultimately refused vaccination. Site 2 was innovative in promoting the flu campaign, creating humorous themes that change annually, and holding a contest for best flu-videos. When we asked the infection-control nurse about the value of this, she said “We make it a topic that employees talk about.” Both Sites 1 and 2 provided an opportunity for personal counselling for employees who refuse the vaccine. Such activities took place during massive vaccine campaigns, allowing the flu team to incorporate targeted material that addresses some of the misconception about the vaccine and apply advanced techniques, such as motivational interviewing, to resolve ambivalence about receiving the vaccine.

Low performing sites simply relied on prepared educational material that was mostly supplied by the national VHA Office of Public Health, and did not develop targeted or humorous materials at the local level. Educational materials used at low performing sites lacked innovation that would engage employees and increase their interest in the flu campaign.

### Strong practice 5: performance audit and feedback by department

Only high-performing sites provided a mechanism to share vaccination rates with employees. At Site 1, department managers receive weekly or bi-weekly updates of vaccination rates in their units, which are shared across the facility. This pressured local managers not only to track vaccination rates but also to urge employees to receive the vaccine. One manager at Site 1 explained “now I’m in a competition too, because all the managers’ units are listed, you don’t want everybody at the medical center to have 99 % and [you have less]”. At Site 3, the Director emphasized feedback as key to his site’s success. “…giving them feedback about how *we* are doing compared to others nationally and also within our [region] is key.”

Low performing sites did not have a process to feed back vaccination rates to unit managers or employees. This may have been expected, since there was no process to track employee vaccination status, which would have been necessary to produce such a report.

## Discussion

In this study, we compared organizational characteristics between VHA sites with consistently high rates of HCP flu vaccination and sites with consistently low rates. Our main findings were that high-performing sites were characterized by supportive executive leadership involvement, positive flu team characteristics, and the presence of five strong practices. Consistent with prior studies, we observed that all campaigns face real barriers to success – even at the high-performing sites. Our study suggests that it is not the absence of such challenges that distinguishes high-performing sites, but rather a more effective approach to overcoming them. This point speaks directly to the value of our study design (“the positive deviance method”), where the solutions that are identified are always within the realm of the possible. Adopting such design highlights existing organization factors that are associated with better solutions to a problem despite encountering similar challenges. While previous studies focused mainly on describing these challengesthe challenges all sites face, our findings advances the field further by identifying successful strategies to overcome themkey factors that must be present to overcome them [7, 8].

While our finding about the importance of executive leaders to a successful vaccination campaign may sound somewhat unsurprising, previous studies provided only limited detail about this factor [9, 10]. In our study, we provided a rich description about how leadership figures are the backbone of any successful improvement effort, a view that has been widely held in many fields of study [16]. We observed the centrality of leaders in conveying a strong message to employees about the importance of the campaign through symbolic actions. They also played a major role in monitoring the progress of the campaign and enabling the necessary resources for its success. Furthermore, our data shows that teams which enjoy leadership support also feel empowered to problem-solve.

Another facilitator of success that we observed was the importance of establishing and investing in collaborative relationships. Not only did strong levels of collaboration exist within the flu team at high performing sites, but we also observed strong collaborative ties across departments and with executive leadership figures.

One of the goals of this study was to evaluate the extent to which different extant guidelines about how to run a campaign are likely to produce the desired result. Thus, we intentionally inquired about practices suggested by the Healthcare Infection Control Practices Advisory Committee (HICPAC) and The Advisory Committee on Immunization Practices (ACIP) [17]. Many of these practices were shared equally among all sites, and did not differentiate high and low performers; including using mobile carts, staff vaccinating each other, and declination forms. However, some features of these guidelines did seem to distinguish between high- and low-performing sites, including planning the campaign in advance, providing wider access to the vaccine, and implementing intense and innovative strategies to promote and deliver the vaccine. Although ACIP recommends “measuring and reporting” flu vaccination rates as a strategy to improve HCP vaccination rates, we noted that measuring and reporting is not sufficient to produce improved results; the high-performing sites also used this information to target efforts to reach unvaccinated employees and to pressure department managers to have their employees vaccinated. Reporting performance back to low-performing sites that have little organizational support (leadership) or organizational structure (empowered and collaborative flu vaccination team) in all likelihood will not result in improved vaccination rates.

Similar to a previous study [18], we observed advantages for using emergency drills as a method to massively vaccinate HCP at the two larger high-performing sites. Combining vaccination with a mandatory drill, where all employees were expected to attend, allowed the team to take advantage of institutional resources that lessened the burdens of documentation and reporting the vaccination status for each employee. It provided opportunities for targeted education for employees, allowing the use of special techniques such as motivational interviewing.

Our study is limited by examining sites within the VHA, a unique integrated health care system. Some aspects of organizational culture and operational realities present within the VHA may not be fully duplicated elsewhere. We might have learned more by studying more than six sites, although we seem to have explored many of the different ways that sites can arrive at high- or low-performance status. Finally, limited funding precluded other qualitative methods such as face to face interviews and ethnographic observation that are commonly used in studying organization characteristics to be integrated in our methods.

## Conclusion

We highlight several recognizable features that are consistently present in high-performing HCP flu campaigns, and absent from low-performing campaigns. Our results can serve as a guide for any site wishing to improve its flu campaign, especially when a mandate is not feasible. Our findings have already been disseminated across VHA sites, with a goal of inducing sites to adopt these best practices. Several sites have already asked for logistical help in making these changes, suggesting that our work is already having an impact. The present publication is intended to disseminate our findings outside the VHA as well.

## Abbreviations

ACIP, Advisory Committee on Immunization Practices; ACOEM, American College of Occupational and Environmental Medicine; HCP, health care providers; HICPAC, Healthcare Infection Control Practices Advisory Committee; IRB, Institutional Board Review; SHEA, Society for Healthcare Epidemiology of America; VHA, Veterans Health Adminstrations

## Additional file

Additional file 1: Interview guide for the key informants of the health care provider influenza vaccination project. (DOCX 18 kb)
