# Supplementary material for: Organizational factors associated with Health Care Provider (HCP) influenza campaigns in the Veterans health care system: a qualitative study
Source: BMC Health Serv Res. 2016 Jul 4;16:211. doi: 10.1186/s12913-016-1462-y (PMC4932695; doi:10.1186/s12913-016-1462-y)
Supplement: Additional file 1: — Interview guide for the key informants of the health care provider influenza vaccination project. (DOCX 18 kb) [file 12913_2016_1462_MOESM1_ESM.docx]

**Interview guide for the key informants of the health care provider influenza vaccination project**

Name and title Position:

Years in Current Position:

Site:

Date of Interview:

Questions

1- Describe your role in the HCP vaccination program.

-What is your role in planning the HCP vaccination program?

-What is your role in implementing the HCP vaccination program?

-What is your role in evaluating the HCP vaccination program?

2- Would you consider yourself a leader in the HCP program?

- Would others consider you a leader/champion of the HCP program?

-How does this role fit in with your other responsibilities?

-What proportion of your time is spent on this program at all different stage of planning, implementation and evaluation?

1. Who else is recognized as being very involved with the program?

-Does the facility have a flu champion? How long has that person been in that position? How often do people in this role change?

1. What is it like to be part of the HCP vaccination campaigns?

-How would you describe your working area during the HCP vaccine season at all stages of HCP campaign?

-Where does the HCP program live (your offices, etc)? Any concerns?

1. Walk me through your daily activities that are related to the HCP program during the vaccine season including activities during planning implementation and evaluation of the HCP program.

-Describe a typical day.

1. Tell me about the staff that works with you during the HCP vaccine program.

-What are their assigned positions during the HCP vaccination program?

-Have these positions turned over recently?

-How well do they work together?

1. What is the timeline for your HCP influenza vaccine campaign for all stages of preparation, implementation and evaluation?

-Does it vary based on the availability of the vaccine?

-Do veterans have any priority receiving the flu vaccine?

1. How many health care providers are you in charge of vaccinating per season?

-How many of health care providers work the health care system?

-Who else do you offer the flu vaccine to?

1. -Does your site combine the vaccination program for Veterans and employees?

-How so?

-Are they planned together?

-Do they share the same timelines, and goals?

-Do they share the same staff and clinics?

1. How many facilities are affiliated with you? (CBOC , campuses, dormitories, etc..)
2. How do you gather information on HCP who get vaccinated outside the VA?
3. Does your facility have policies or recommendations regarding HCP vaccination? If so, please describe
4. How do you promote your HCP flu vaccination campaign?

-Do you publicize it through email, posters, flyers, or other means?

- Do you promote it as a patient safety measure?

1. What happens if an employee does not get a flu vaccine or refuses?

-Are they required to explain the reason why they did not receive it?

-Do you keep statistics on the reasons for declining?

-Are they offered additional information/education?

-Do you require a signed form to show that?

-How do you know if a HCP got a flu shot outside of VA?

-How do you document this? What do you document about it?

1. What do you know about the employees who do not get vaccinated?

-Do you have any anecdotal evidence about who doesn’t get vaccinated and why they don’t?

-If you have a brief example that would be great. (my questions are optional—depends on time and they are also another way of asking for the same information)

1. Do you have any sense of what promotion method is more successful/ effective than others to encourage HCP vaccination?

-What do you think works best and why?

-Does it make any difference for different groups of HCP (nurses, medical assistants, etc)?

1. How do members of your vaccine program communicate with each other? (Meetings, emails, etc.)

-If meetings, are they scheduled, and how often?

-Does this vary based on time of year?

1. How do you address problems or challenges with your campaign?

-How do you troubleshoot a problem? Give me an example.

-How could that problem have been solved better?

1. How important is the HCP vaccine program in the eyes of:

-Your immediate supervisor.

-The chief of staff or the hospital administration at your institution? Please give examples.

1. What is the role of the unions in ensuring a successful campaign?
2. How do you ensure that night shift employees (or off-tour employees) are offered the vaccine?
3. How would describe the resources that are available to you? What more would you need?

-what would extra money achieve?

-what would extra staff achieve?

-What would extra time achieve?

- What would more equipment/supplies achieve?

More training?

More educational resources and materials?

1. Tell me about performance measurement for your HCP vaccination program.

-What do you think is an acceptable vaccination rate?

- What is your facility vaccination rate?

-How do you know this information?

If you aren’t satisfied with the HCP rates of vaccination, what will you do

differently? Who determines these actions?

1. Who looks at the performance of your HCP vaccination program? Does it get reported to anyone inside or outside your Hospital? (If yes, to whom)
2. What are some practices that work well in your HCP vaccination program? Tell me why you think they are effective?
3. What are the areas that need improvement in your HCP vaccination program? What concerns you about your program? Why so?
4. How do you think last season's campaign was different from the previous ones?
5. How will next season be different?

30 -Imagine getting all your wishes to improve the HCP vaccine program. What would you do?

-What resources, staff, materials, and equipment would you like?

Missing points

Can you think of anything that I did not ask, but should have? Is there anything you think I should know about that is specific to your location and to your HCP program? Who else would be a good source of information? Is there anyone else I should speak to?
